# The Two-Component Signal Transduction System CopRS of *Corynebacterium glutamicum* Is Required for Adaptation to Copper-Excess Stress

**DOI:** 10.1371/journal.pone.0022143

**Published:** 2011-07-20

**Authors:** Stephanie Schelder, Daniela Zaade, Boris Litsanov, Michael Bott, Melanie Brocker

**Affiliations:** Institut für Bio-und Geowissenschaften, IBG-1: Biotechnologie, Forschungszentrum Jülich, Jülich, Germany; Monash University, Australia

## Abstract

Copper is an essential cofactor for many enzymes but at high concentrations it is toxic for the cell. Copper ion concentrations ≥50 µM inhibited growth of *Corynebacterium glutamicum*. The transcriptional response to 20 µM Cu^2+^ was studied using DNA microarrays and revealed 20 genes that showed a ≥ 3-fold increased mRNA level, including cg3281-cg3289. Several genes in this genomic region code for proteins presumably involved in the adaption to copper-induced stress, e. g. a multicopper oxidase (CopO) and a copper-transport ATPase (CopB). In addition, this region includes the *copRS* genes (previously named *cgtRS9*) which encode a two-component signal transduction system composed of the histidine kinase CopS and the response regulator CopR. Deletion of the *copRS* genes increased the sensitivity of *C. glutamicum* towards copper ions, but not to other heavy metal ions. Using comparative transcriptome analysis of the Δ*copRS* mutant and the wild type in combination with electrophoretic mobility shift assays and reporter gene studies the CopR regulon and the DNA-binding motif of CopR were identified. Evidence was obtained that CopR binds only to the intergenic region between cg3285 (*copR*) and cg3286 in the genome of *C. glutamicum* and activates expression of the divergently oriented gene clusters cg3285-cg3281 and cg3286-cg3289. Altogether, our data suggest that CopRS is the key regulatory system in *C. glutamicum* for the extracytoplasmic sensing of elevated copper ion concentrations and for induction of a set of genes capable of diminishing copper stress.

## Introduction

Due to its ability to change between the oxidised Cu^2+^ and reduced Cu^+^ state, copper has become a versatile cofactor for enzymes involved in electron transport or redox reactions such as cytochrome *c* oxidases or monooxygenases [1]. However, in high concentrations uncomplexed copper ions can generate reactive oxygen species or lead to sulfhydryl depletion and thereby become toxic for the cell [2]. Hence, the amount of copper ions inside the cell must be tightly regulated to prevent deprivation as well as high, toxic copper concentrations. In prokaryotes several copper resistance systems have been characterised, among the best studied systems being those of *Enterococcus hirae* for Gram-positive and of *Escherichia coli* for Gram-negative bacteria (for reviews see [2]–[5]).

In *E. hirae* the *cop* operon is mainly responsible for copper homeostasis. It consists of four genes coding for a transcriptional repressor (CopY), a copper chaperon (CopZ) and two copper P-type ATPases (CopA and CopB). In the presence of elevated copper concentrations CopZ donates Cu^+^ to CopY resulting in a derepression of the *cop* operon and subsequently in copper export by CopB [2]. In *E. coli*, copper homeostasis is achieved by the action of the MerR-type regulator CueR in concert with two-component systems, such as the CusRS two-component system [4], [6], [7]. In the presence of elevated copper concentrations, CueR activates the transcription of *copA* and *cueO* encoding a P-type ATPase and an oxygen-dependent multicopper oxidase, respectively [8], [9]. CopA is responsible for exporting excess Cu^+^ from the cytoplasm into the periplasm where it is oxidised to the less toxic Cu^2+^ by CueO. The two-component system CusRS was found to play a role in copper homeostasis under anoxic conditions. It represents a prototypical two-component system [10] where the membrane-bound sensor kinase CusS monitors the periplasmic copper concentration and autophosphorylates a histidine residue at elevated copper concentrations. The phosphoryl group is then transferred to an aspartate residue of the response regulator CusR, which then activates transcription of the *cusRS* operon and of the adjacent but divergently oriented *cusCFBA* operon [11]. The translation products CusCBA (a proton-cation antiporter) and CusF (a copper chaperone) then contribute to copper tolerance under copper stress conditions.

Recently a novel type of copper-sensing transcriptional repressors (CsoR-type) was identified in *Mycobacterium tuberculosis* [12]. In *M. tuberculosis*, when there is no copper excess stress, the transcriptional regulator CsoR represses the expression of its own operon (*cso* operon) which includes a gene coding for the putative copper exporter CtpV [13], [14]. By binding Cu^+^, CsoR loses its DNA-binding affinity resulting in derepression of the *cso* operon and export of copper *via* CtpV.

In the soil bacterium *Corynebacterium glutamicum* [15], [16], a close relative of *M. tuberculosis*, the control of copper homeostasis has not been studied yet. *C. glutamicum* belongs to the *Corynebacterium-Mycobacterium-Nocardia* group of actinomycetes and serves as a nonpathogenic model organism for studying selected features common to corynebacteria and pathogenic mycobacteria. Additionally, this species is of interest due to its biotechnological importance as a producer of L-glutamate and L-lysine. Recent studies suggested that *C. glutamicum* possesses four cuproproteins (the cytochrome *aa*_3_ oxidase subunits I and II and two multicopper oxidases), two copper transporters which are likely exporters, and four copper chaperones [1], [17], [18]. Since cytochrome *aa*_3_ oxidase plays a key role in the energy household of *C. glutamicum* [19], copper homeostasis is probably also important for biotechnological production processes.

Here we investigated copper homeostasis and its regulation in *C. glutamicum*. We identified the copper excess stimulon and provided evidence that the adaption to excess copper involves the two-component signal transduction system CopRS (previously named CgtRS9, Accession UniProtKB Q6M1P4 and Q8NLH8). We could identify direct target genes of the response regulator CopR and show that these were activated in a copper-dependent manner. The CopR DNA-binding motif was identified and evidence was provided that CopR is active in its phosphorylated state.

## Results

### Response of *C. glutamicum* to elevated copper concentrations

**Figure 1:**
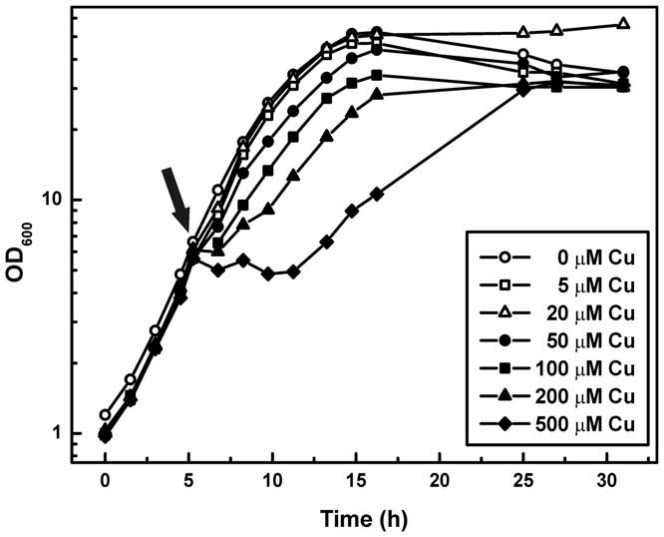
Influence of different copper ion concentrations on the growth of *C. glutamicum* wild type. Cells pregrown in CGXII minimal medium with 4% (w/v) glucose were used to inoculate 50 ml of fresh CGXII medium (1.25 µM CuSO_4_). When the cultures had reached an OD_600_ of about 5–6, different CuSO_4_ concentrations were added (indicated by an arrow). The cultures were incubated at 30°C on a rotary shaker at 120 rpm. The growth curves shown here are representative of those from three independent growth experiments with comparable results.

First, the growth of *C. glutamicum* wild type in the presence of elevated copper ion concentrations was determined. Therefore, cells were grown in CGXII minimal medium (standard copper concentration: 1.25 µM) to an OD_600_ of 5–6 and then different CuSO_4_ concentrations (5–500 µM) were added to the cultures (Fig. 1). Whereas the addition of 5 and 20 µM CuSO_4_ had no effect on the growth of *C. glutamicum* compared to the control culture (no additional copper), higher CuSO_4_ concentrations led to reduced growth rates. The addition of 500 µM CuSO_4_ completely inhibited growth of *C. glutamicum*, however, after approx. 7 h the culture resumed growth and reached the usual final cell density.

In order to identify genes that were differentially expressed in the presence of elevated copper ion concentrations in the medium, DNA microarray experiments were performed. *C. glutamicum* wild type cells were pre-cultivated in CGXII minimal medium overnight and then used to inoculate fresh standard CGXII medium (containing 1.25 µM CuSO_4_) or CGXII medium containing 21.25 µM CuSO_4_. After the cultures had reached an OD_600_ of 5–6, the cells were harvested and used for RNA preparation. Altogether 26 genes showed a more than threefold changed mRNA level in at least two of four independent biological replicates (*P*-value≤0.05). Six of these genes, all of which encode proteins of unknown function, showed a decreased mRNA level and 20 genes an increased mRNA level in the presence of 21.25 µM CuSO_4_ (Table 1). The latter group included two genes coding for the transcriptional regulators ArsR1 and CopR (previously named CgtR9). ArsR1 belongs to the SmtB/ArsT family of metal-sensing transcriptional repressors and regulates the *ars* operons (cg0318-cg0319 and cg1705-cg1707) in an arsenic-dependent manner [20]. In the presence of As^3+^, a derepression of the *ars* operons occurs which leads to increased tolerance to elevated arsenic concentrations. Despite the increased mRNA level of *arsR1* the expression level of the *ars* operons was not altered, indicating that an elevated copper ion concentration does not cause a dissociation of ArsR1 from its target promoters and hence no derepression of the target genes. CopR is a response regulator and part of the CopR-CopS two-component system [21]. The *copRS* genes (cg3285 and cg3284, respectively) as well as the up- and downstream genes (cg3286-cg3289 and cg3283-cg3281) exhibited highly increased mRNA levels (Table 1), indicating that this gene region is particularly important for the adaption to copper excess conditions. This assumption is supported by the fact that several of these genes code for proteins that are obviously linked to copper homeostasis, such as a putative copper-transporting ATPase (CopB, Cg3281; Accession UniProtKB Q8NLI0) and a secreted multicopper oxidase (CopO, Cg3287; Accession UniProtKB Q8NLH5). Since genes encoding transcriptional regulators are often localised in the immediate vicinity of their target genes, the CopRS two-component system might be responsible for the altered expression of cg3281-cg3289.

**Table 1:** Transcriptome comparison of *C. glutamicum* ATCC 13032 cultivated in CGXII minimal medium supplemented with 21.25 µM CuSO_4_ (Cu^↑^) and in standard CGXII medium with 1.25 µM CuSO_4_ (Cu^s^) using DNA microarrays.

cg no.	NCgl no.	Gene	Known or predicted function of gene product	Cu^↑^/Cu^s^
**Copper-related proteins**				
cg3281	NCgl2859	*copB*	probable cation-transporting ATPasetransmembrane protein	11.22
cg3282	NCgl2860		heavy metal binding transport protein	14.91
cg3283			protein of unknown function	12.36
cg3284	NCgl2862	*copS*	two component sensor kinase	545.93
cg3285	NCgl2863	*copR*	two component response regulator	38.68
cg3286	NCgl2864		secreted protein of unknown function	52.96
cg3287	NCgl2865	*copO*	secreted multicopper oxidase	267.14
cg3288			protein of unknown function	422.20
cg3289	NCgl2866	*tlpA*	thioredoxin-like protein	235.59
**Proteins involved in heme biosynthesis and cytochrome ** ***c*** ** maturation**				
cg0518	NCgl0422	*hemL*	glutamate-1-semialdehyde-2,1-aminomutase	3.63
cg0519	NCgl0423		putative phosphoglycerate mutase	4.13
cg0520	NCgl0424		periplasmic thioredoxin	4.62
cg0522	NCgl0425	*ccsA*	cytochrome *c* biogenesis protein membrane protein	3.61
cg0524	NCgl0427	*ccsB*	cytochrome *c* assembly membrane protein	3.65
**Other proteins**				
cg0915	NCgl0769	*ftsX*	putative cell division protein	3.79
cg1109	NCgl0933	*porB*	anion-specific porin precursor	3.61
cg1704		*arsR1*	ArsR-type transcriptional regulator	6.02
**Proteins of unknown function**				
cg0905	NCgl0760		secreted protein of unknown function	0.19
cg1514	NCgl1289		protein of unknown function	0.32
cg1918	NCgl1635		secreted protein of unknown function	0.32
cg2058			protein of unknown function	0.25
cg2348	NCgl2059		lipoprotein of unknown function	4.55
cg2799	NCgl2452		secreted protein of unknown function	3.88
cg3343	NCgl2912		secreted membrane protein of unknown function	0.31
cg3344	NCgl2913		protein of unknown function	52.92
cg4005	NCgl1288		lipoprotein of unknown function	0.22

The mRNA ratios shown represent mean values from four independent DNA microarray experiments starting from independent cultures. The wild type was cultivated in CGXII minimal medium with 4% (w/v) glucose with or without additional 20 µM CuSO_4_ and mRNA was prepared from cells in the exponential growth phase. The table includes those genes which showed an at least threefold changed mRNA level (increased or decreased) in at least two of the four replicates with a *P*-value of ≤0.05.

In summary, the transcriptome data led to the identification of the copper excess stimulon and provided evidence for the involvement of CopR in the regulation of copper homeostasis in *C. glutamicum*. In the remaining part of this study the role of the CopRS two-component system in copper homeostasis was investigated.

### Influence of CopRS on the resistance to heavy metals

**Figure 2:**
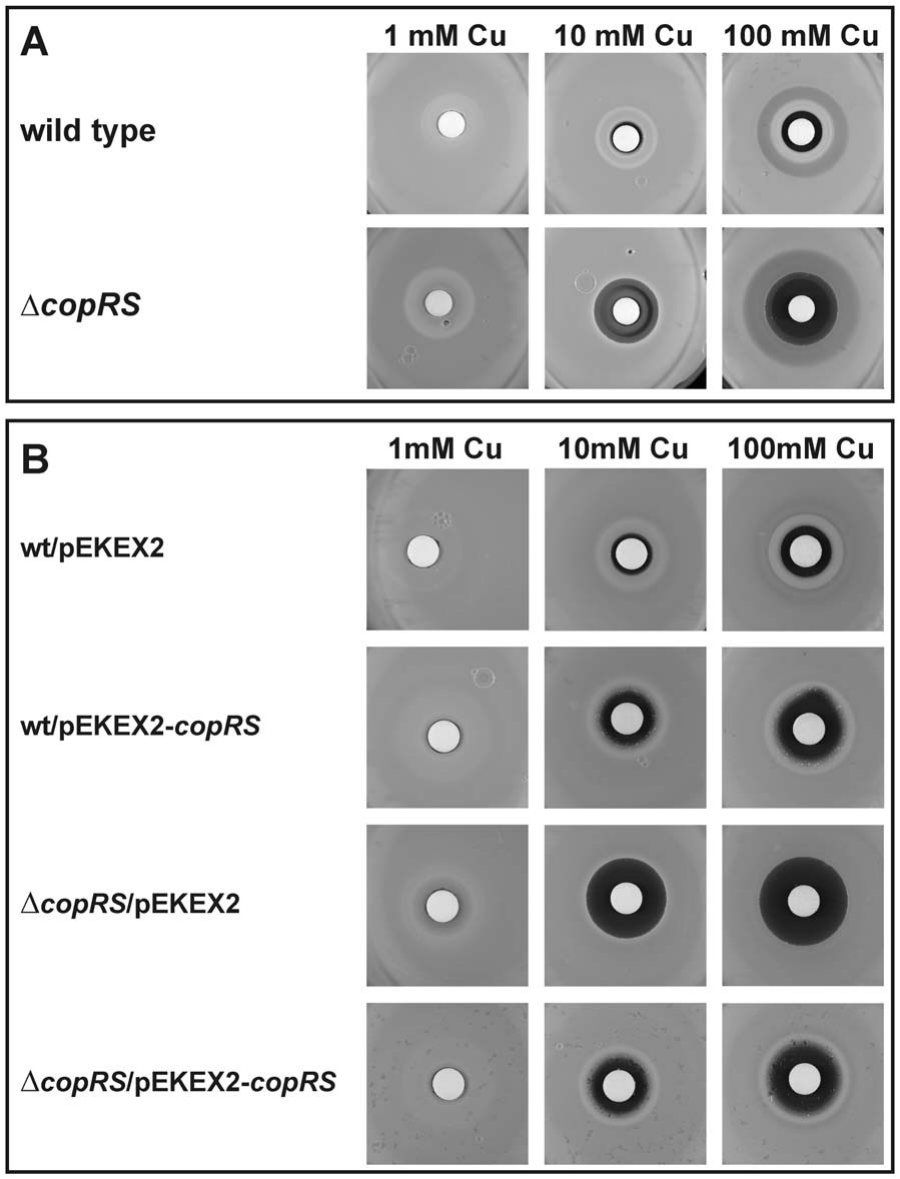
Agar diffusion assays showing growth inhibition of different *C. glutamicum* strains by copper ions. (A) Comparison of the copper sensitivity of *C. glutamicum* wild type and the Δ*copRS* deletion mutant. The inhibition zone (black halo) increased with higher copper concentrations and was larger for the deletion mutant. (B) The *copRS* deletion can be complemented using the plasmid encoded *copRS* genes. For experimental details see Material and Methods.

**Figure 3:**
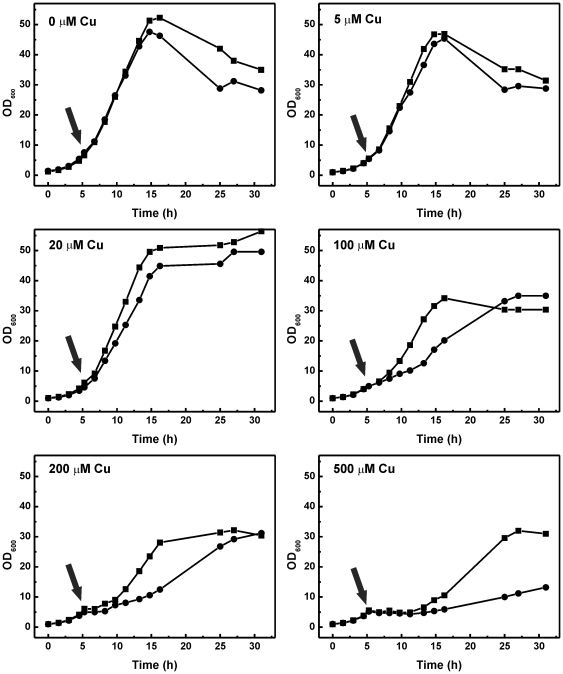
Influence of increasing copper ion concentrations on growth of *C. glutamicum* wild type (▪) and *C. glutamicum* Δ*copRS* (•). For experimental details see legend to Fig. 1. The time points of CuSO_4_ addition are indicated by arrows.

In order to verify the involvement of the CopRS two-component system in copper homoeostasis, the resistance of a *copRS* deletion mutant [21] to copper ions and other heavy metal salts was compared to the wild type using agar diffusion assays. An increased susceptibility was only observed for copper ions (Fig. 2A) but not for all other heavy metal ions tested (nickel, manganese, zinc, silver, cobalt, lead or cadmium; data not shown). Cultivation of Δ*copRS* and *C. glutamicum* wild type in CGXII minimal medium to which different CuSO_4_ concentrations (5–500 µM) were added when the cultures had reached an OD_600_ of 5–6 corroborated the reduced resistance to copper ions of the mutant strain (Fig. 3). The phenotype could be complemented by plasmid-borne *copRS* expression (Fig. 2B). These results confirmed the assumption that CopRS is involved in copper homeostasis of *C. glutamicum*.

### Comparison of the expression profiles of the Δ*copRS* mutant and the wild type

To identify the regulon of the response regulator CopR, the transcriptome of the Δ*copRS* deletion mutant was compared to that of the wild type using DNA microarrays. For cells grown in standard CGXII medium (1.25 µM CuSO_4_), no significant gene expression differences were observed, indicating that the CopRS two-component system is not active under this condition (data not shown). However, in the presence of elevated copper ion concentrations (21.25 µM), the mRNA level of 43 genes was changed more than threefold (Table 2) in the Δ*copRS* mutant, showing that CopRS is active in the presence of elevated copper concentrations and further supporting our hypothesis that CopRS is involved in copper-induced stress regulation. Besides *copS* and *copR*, the strongest downregulation was observed for the gene cluster cg3286-cg3289 (50- to 100-fold decreased mRNA level in the mutant strain), which is located directly upstream of *copRS* in reverse orientation and belongs to the copper excess stimulon. The genes encode a secreted protein of unknown function (Cg3286), a putative secreted multicopper oxidase (CopO, Cg3287), a protein of unknown function (Cg3288), and a thioredoxin-like protein (TlpA, Cg3289). In addition, several genes which encode transport systems were downregulated in the mutant (Table 2). Except for the glutamate uptake system (GluABCD), none of these transport systems has been characterised in detail yet, but it was supposed that Cg0507 and Cg0508, Cg0924-Cg0927, and Cg3434 are involved in iron uptake [22], [23]. The genes cg3281-cg3283, which were also part of the copper excess stimulon, showed a 1.6-2.3-fold (*P*-value≤0.05) decreased mRNA level in the Δ*copRS* mutant, as well (data not shown).

**Table 2:** Transcriptome comparison of *C. glutamicum* wild type (wt) and the Δ*copRS* deletion mutant after cultivation in CGXII minimal medium supplemented with 21.25 µM CuSO_4_ (Cu^↑^) using DNA microarrays.

cg no.	NCgl no.	Gene	Known or predicted function of gene product	Δ*copRS* Cu^↑^/wt Cu^↑^
**Copper-related proteins**				
cg3284	NCgl2862	*copS*	two component sensor kinase	0.00
cg3285^†^	NCgl2863	*copR*	two component response regulator	0.00
cg3286^†^	NCgl2864		secreted protein of unknown function	0.01
cg3287^†^	NCgl2865	*copO*	secreted multicopper oxidase	0.01
cg3288			protein of unknown function	0.01
cg3289^†^	NCgl2866	*tlpA*	thioredoxin-like protein	0.02
**Transporter or transport-related proteins**				
cg0507	NCgl0412		ABC-type transporter, permease component	0.30
cg0508^†^	NCgl0413		secreted substrate-binding lipoprotein	0.29
cg0622	NCgl0510		ABC-type cobalt transport system, ATPase component	0.24
cg0623	NCgl0511		ABC-type cobalt transport system, permease component	0.26
cg0624^†^	NCgl0512		secreted oxidoreductase; protein of unknown function	0.25
cg0924^†^	NCgl0776		secreted siderophore-binding lipoprotein	0.20
cg0926^†^	NCgl0777		siderophore ABC transporter, permease protein	0.30
cg0927	NCgl0778		siderophore ABC transporter, permease protein	0.30
cg2136^†^	NCgl1875	*gluA*	glutamate uptake system ATP-binding protein	0.38
cg2137	NCgl1876	*gluB*	secreted glutamate binding protein	0.37
cg2138	NCgl1877	*gluC*	glutamate permease	0.39
cg2181^†^	NCgl1915		ABC-type peptide transport system, secreted component	0.24
cg2182	NCgl1916		ABC-type peptide transport system, permease component	0.22
cg2183	NCgl1917		ABC-type peptide transport system, permease component	0.20
cg2184	NCgl1918		ABC-type peptide transport system, ATPase component	0.19
cg2610^†^	NCgl2294		ABC-type transport system, secreted component	0.34
cg3320^†^	NCgl2891		ABC-type transporter, permease component	0.20
cg3404^†^	NCgl2970		secreted siderophore-binding lipoprotein	0.26
**Other**				
cg0414^†^	NCgl0337	*wzz*	cell surface polysaccharide biosynthesis/chain length determinant protein	0.25
cg0424^†^	NCgl0347		putative glycosyltransferase	0.09
cg0797	NCgl0665	*prpB1*	methylisocitric acid lyase	3.16
cg0998	NCgl0841		trypsin-like serine protease	3.53
cg1055	NCgl0888	*menG*	ribonuclease activity regulator protein RraA	3.24
cg1290	NCgl1094	*metE*	5-methyltetrahydropteroyltriglutamate-homocysteine methyltransferase	0.29
cg1487	NCgl1262	*leuC*	isopropylmalate isomerase, large subunit	0.37
cg2925	NCgl2553	*ptsS*	enzyme II sucrose protein	0.38
cg3226^†^	NCgl2816		putative L-lactate permease	0.02
**Proteins of unknown function**				
cg0077^†^	NCgl0057		hypothetical protein	11.67
cg0078	NCgl0058		hypothetical protein	10.02
cg0416	NCgl0339		secreted protein	0.34
cg0625	NCgl0513		secreted protein	0.29
cg1326^†^	NCgl1126		hypothetical protein	0.31
cg2799^†^	NCgl2452		secreted protein	3.29
cg3009			hypothetical protein	0.27
cg3213	NCgl2805		secreted protein	3.71
cg3377^†^	NCgl2945		hypothetical protein	8.76
cg3378^†^	NCgl2946		hypothetical protein	11.29

The mRNA ratios shown represent mean values from five independent DNA microarray experiments starting from independent cultures. The strains were cultivated in CGXII minimal medium with 4% (w/v) glucose with additional 20 µM CuSO_4_ and mRNA was prepared from cells in the exponential growth phase. The table includes those genes which showed an at least threefold changed mRNA level (increased or decreased) in at least three of the five replicates with a *P*-value of ≤0.05. The putative promoter regions of the genes indicated with a ^†^ were chosen for the EMSAs (see Fig. S1).

### Search for direct target genes of CopR

**Figure 4:**
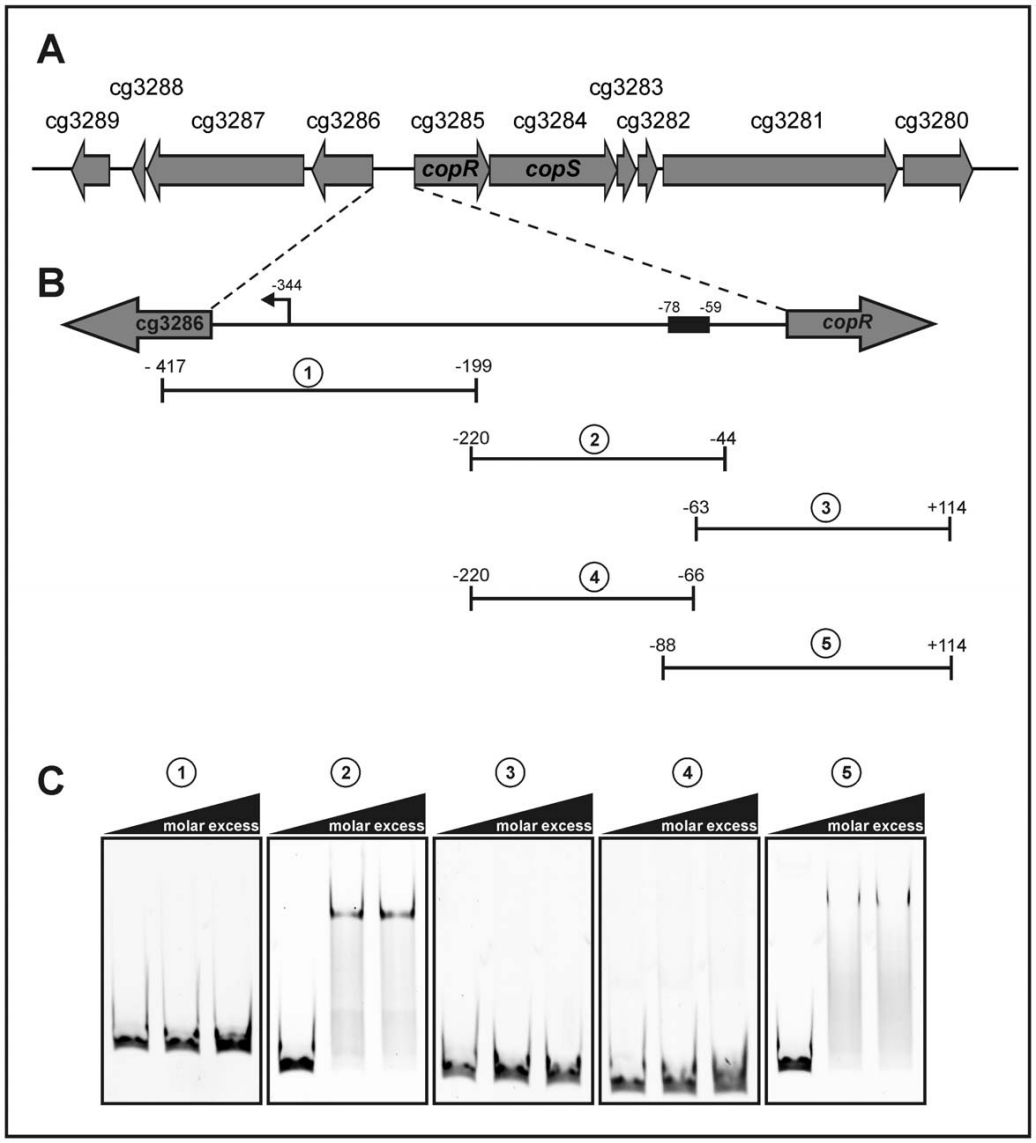
Search for the CopR-binding motif within the cg3286-*copR* intergenic region. (A) Scheme of the genome region covering cg3280-cg3289 based on the CoryneRegNet annotation (http://coryneregnet.cebitec.uni-bielefeld.de). The genes code for a secreted protein of unknown function (cg3280), a probable cation-transporting ATPase transmembrane protein (*copB*, cg3281), a heavy metal binding transport protein (cg3282), a protein of unknown function (cg3283), a secreted protein of unknown function (cg3286), a secreted multicopper oxidase (*copO*, cg3287), a protein of unknown function (cg3288) and a thioredoxin-like protein (*tlpA*, cg3289). (B) Enlargement of the cg3286-*copR* intergenic region and position of DNA fragments 1-5 used for EMSAs. The numbers above the DNA fragments indicate the bp distance of their ends to the translation start site of *copR*. (C) EMSAs with fragments 1–5 (100 nM, 155–219 bp in length) and phosphorylated CopR. The molar excesses of the CopR used were 0-, 25-, 50-fold.

In order to test which of the genes with an altered expression in the Δ*copRS* mutant are direct target genes of the response regulator CopR, electrophoretic mobility shift assays (EMSAs) were performed with purified CopR and the relevant promoter regions. As phosphorylated CopR protein was found to have a higher binding affinity compared to unphosphorylated CopR (see below), all gel shift experiments were performed with CopR which had been preincubated with the low molecular weight phosphoryl donor acetylphosphate. In a first set of experiments, 21 promoter regions of putative CopR target genes (see Table 2, indicated with ^†^) and as a negative control the promoter region of an arbitrarily chosen gene (cg1665) were tested. As shown in Fig. S1, binding of CopR was only observed with the DNA fragment containing the promoter region of *copR* (229 bp, +9 to −220 corresponding to the *copR* translational start site). A complete retardation was observed in the presence of a 25-fold molar excess of phosphorylated CopR. Since the putative binding site of CopR is located in the intergenic region of *copR* and cg3286, which are orientated divergently (Fig. 4A), not only the cg3285-cg3281 genes, but also the cg3286-cg3289 genes might be regulated by CopR.

### Influence of phosphorylation on CopR binding affinity

To analyse the influence of phosphorylation on the DNA-binding properties of the response regulator CopR, the apparent *K_d_* value of unphosphorylated CopR and of CopR that had been preincubated with acetylphosphate were determined using a 5′-Cy3-labelled DNA fragment (90 bp) that covers the DNA region from −44 to −110 upstream of the *copR* translational start site (Fig. S2). For CopR preincubated with acetylphosphate an apparent *K_d_* value of 0.4 µM was calculated (CopR concentration required for binding half of the DNA). Unphosphorylated CopR exhibited a significantly lower affinity and showed an apparent *K_d_* value of 2.3 µM. These results indicate that CopR is phosphorylated by acetylphosphate and that phosphorylation increases the DNA-binding affinity.

### Identification of the CopR binding site

In order to identify the CopR binding motif, EMSAs with subfragments of the intergenic region between cg3286 and *copR* were performed. The subfragments were incubated with phosphorylated CopR at two different molar excesses (25- and 50-fold) and separated by 10% native PAGE. As shown in Fig. 4B and C, binding was observed for fragments 2 and 5, but not for fragments 1, 3, and 4. Consequently, a CopR binding site must be localised between bp −59 and −78 with respect to the *copR* translational start site. This corresponds to position −266 and −285 with respect to the cg3286 transcriptional start site, which had been mapped by primer extension analysis 59 bp upstream of the cg3286 start codon (Fig. S3). The determination of the *copR* transcriptional start site failed so far although different methods were tested.

**Figure 5:**
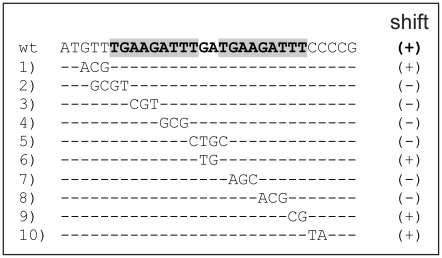
Mutational analysis of the putative CopR binding site within the cg3286-*copR* intergenic region. To determine the importance of the predicted binding motif (sequence in the grey boxes) for CopR binding, fragments with different mutations were tested in EMSAs with phosphorylated CopR. According to the results of the shifts, the fragments were classified into two categories: +, mutated fragment shifted like the wild type fragment; -, the fragment was not shifted.

Analysis of the identified DNA region with the motif alignment and search tool MAST (http://meme.sdsc.edu/meme4_4_0/cgi-bin/mast.cgi) revealed the presence of a perfect direct repeat separated by a two base pair linker (TGAAGATTTgaTGAAGATTT). The relevance of this direct repeat for CopR binding was tested by EMSAs with DNA fragments in which two to four nucleotides of the motif or of the surrounding sequence were exchanged (Fig. 5). Mutations in the motifs' flanking sequence or in the linker did not affect CopR binding, whereas mutations of three base pairs within the binding motif led to a complete inhibition of CopR binding. These results confirmed that the predicted DNA motif is relevant for CopR binding.

To identify further CopR target genes, the binding motif TGAAGATTTnnTGAAGATTT was used to search for similar sequences in the whole *C. glutamicum* genome using the ERGO™ bioinformatics suite (Integrated Genomics, Illinois, USA) allowing four mutations, no deletions and no insertions. 46 hits were found, but only in six cases the putative CopR binding site was located in intergenic regions up to 200 bp upstream of the start codon of the neighbouring gene (cg1336, cg2976, cg3187, cg3337, cg3357 and cg0414). Only one of these genes (cg0414) showed an altered mRNA level in the DNA microarrays comparing Δ*copRS* with the wild type (Table 2). However, no binding of CopR to the promoter region of cg0414 was observed in EMSAs (see Fig. S1), indicating that cg0414 is not a direct target gene of CopR.

### CopR regulation of the *copR* and cg3286 gene

**Figure 6:**
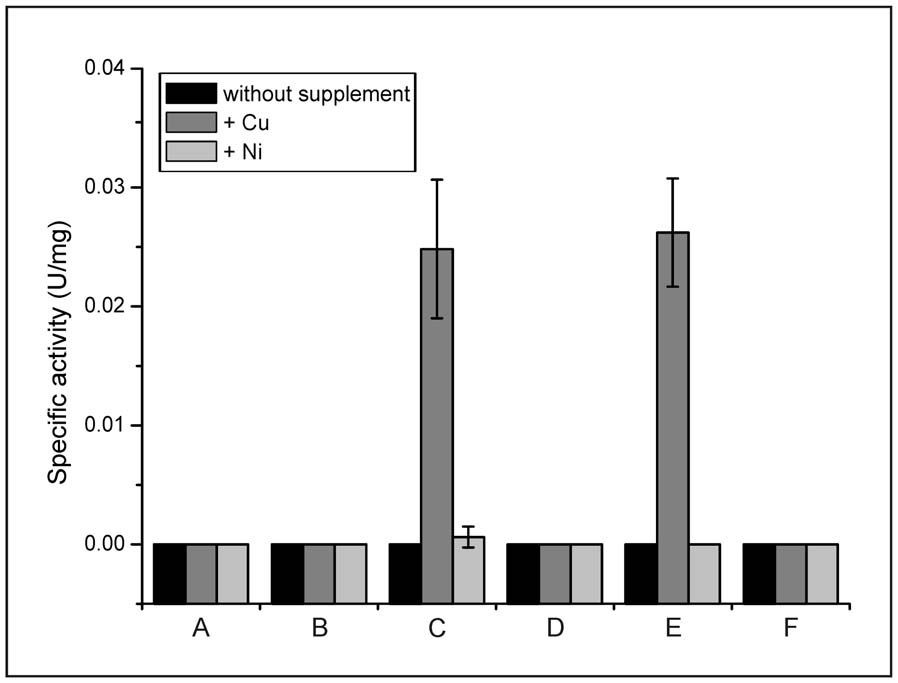
Specific Cat activities of *C. glutamicum* wild type (wt) and the Δ*copRS* mutant. Both strains carrying either the pET2, the pET2-IGR (*copR* promoter) or the pET2-IGR_inverse (cg3286 promoter) vector. Cat activities were measured after growth in CGXII medium containing either 1.25 µM CuSO_4_ (black bars), 20 µM additional CuSO_4_ (dark grey bars) or 20 µM additional NiSO_4_ (light grey bars). A, wt/pET2; B, Δ*copRS*/pET2; C, wt/pET2-IGR (*copR* promoter); D, Δ*copRS*/pET2-IGR; E, wt/pET2-IGR_inverse (cg3286 promoter); F, Δ*copRS*/pET2-IGR_inverse. The values represent averages and standard deviations of three biological replicates.

The data reported above showed that CopR binds to the intergenic region between cg3286 and *copR*, but it was still unclear if CopR activates expression of only the *copR* promoter, of only the cg3286 promoter or of both promoters. To answer this question, reporter gene assays were performed. The plasmids pET2-IGR and pET2-IGR_inverse, which contain the cg3286-*copR* intergenic region in both orientations upstream of a promoterless chloramphenicol acetyltransferase (*cat*) gene, were transferred into *C. glutamicum* wild type and the Δ*copRS* mutant. The resulting strains and the control strains carrying the vector pET2 were cultivated in CGXII minimal medium with or without the addition of 20 µM CuSO_4_ or 20 µM NiSO_4_, respectively. When cultivated in standard CGXII medium with 1.25 µM CuSO_4_ (Fig. 6), none of the strains exhibited Cat activity. Same results were observed when NiSO_4_ was added (Fig. 6). Thus, CopR did not activate transcription of the reporter gene under these conditions. When the medium was supplemented with 20 µM CuSO_4_ (Fig. 6), Cat activity was measurable for the wild type strain harbouring the plasmid pET2-IGR or pET2-IGR_inverse, but not for the Δ*copRS* mutant harbouring one of these plasmids. Also the control strains carrying pET2 had no measurable Cat activity. These results showed that both the cg3286 promoter and the *copR* promoter are activated by CopRS in a strictly copper-dependent manner.

## Discussion

Copper-dependent organisms have evolved sophisticated regulatory mechanisms to ensure a sufficient supply of copper ions on the one hand and a resistance against high concentrations on the other. To enable the cell to react to copper-induced stress, several copper ion sensors were evolved like CueR of *E. coli*, CopY of *E. hirae* and CsoR of *M. tuberculosis* [2], [4], [12], [24]. Besides these cytoplasmic one-component regulators, two-component regulatory systems were identified which are involved in the sensing of elevated copper ion concentrations in the periplasm. Examples are the CusRS system of *E. coli* [7], [11] and the CinSR system of *Pseudomonas putida* [25].

In the present work we studied the response of *C. glutamicum* to elevated copper ion concentrations and identified a novel copper-responsive two-component system, CopRS, which is the key regulatory system for copper ion resistance in this bacterium. The sensor kinase CopS shows the typical domain structure of two-component sensor kinases. The intracellular N-terminus is followed by two predicted transmembrane helices (TMHHM Server: http://www.cbs.dtu.dk/services/TMHMM/) connected via a short periplasmic loop (31 aa). The cytoplasmatic C-terminus harbours a HAMP domain, a HisKA domain and a HATPase_c domain (SMART: http://smart.embl-heidelberg.de/). The sensor kinase CopS of *C. glutamicum* shows 21% and 22% sequence identity to CusS of *E. coli* and CinS of *P. putida*, respectively. As expected, the C-terminal catalytic domains are highly similar but the periplasmic loop of *C. glutamicum* CopS differs from the others since it is much shorter. Within the periplasmic loop of CinS two histidine residues (H37, H147) were identified as potential copper binding site [25]. These two histidine residues are also conserved in CusS but not in CopS. Nevertheless CopS harbours three histidine (H39, H41, H56) and two methionine residues (M42, M44) within its periplasmic loop that might play a role in copper recognition/binding. The response regulator CopR exhibits 33% and 34% sequence identity to *E. coli* CusR and *P. putida* CinR, respectively.

*C. glutamicum* wild type grows unaffectedly up to 20 µM CuSO_4_ (Fig. 1) which is similar to the copper tolerance of *M. tuberculosis*, which grows normally up to 50 µM copper [14] and to that of *E. coli* [7], while it is much lower than that of bacteria like *E. hirae* and *Pseudomonas aeruginosa*, which can grow at copper concentrations up to approx. 8 mM [2], [26].

DNA microarray analyses indicated the involvement of the two-component system CopRS in copper homeostasis (Table 1). This was confirmed by copper sensitivity tests on agar plates and by growth experiments in the presence of different copper concentrations (Fig. 2 and 3). Our approaches revealed two gene clusters regulated by CopR, cg3286-cg3289 and cg3285-cg3281 with cg3285 and cg3284 coding for CopR and CopS, respectively. The translation products of these genes possibly play a role in copper resistance. For example the multicopper oxidase CopO (Cg3287) can detoxify Cu^+^ by converting it to the less toxic Cu^2+^ and by binding free Cu ions while the putative copper transporting P-type ATPase CopB (Cg3281) most likely functions as a copper export pump.

The determined apparent *K_d_* value of 0.4 µM for the phosphorylated form of CopR is in the same range as described for other response regulators [27]. The binding site (TGAAGATTTnnTGAAGATTT) of CopR within the cg3286-*copR* intergenic region (Fig. 4) seems to be the only one within the entire genome of *C. glutamicum.* This situation is not unusual for copper regulatory systems, as *e.g.* the *E. coli* CusRS system also only activates the transcription of its own genes and the divergently orientated *cusCFB* operon [6]. Binding of CopR to this single binding site results in transcriptional activation of both divergently located gene regions (*copR*-cg3281 and cg3286-cg3289). Further the activation is depended on the existence of the CopRS system and this system by itself is only active in the presence of elevated copper ion concentrations (Fig. 6). Since the binding motif is rather far upstream of the transcriptional start site of cg3286 (−266 to −285 bp) the question arises how these genes can be under the control of CopR.

The *cop* gene region (cg3281-cg3289) exhibits an extraordinary high G+C content in comparison to the rest of the genome and is highly conserved in *Corynebacterium diphtheriae* (more than 95% identical) [15], [16]. This high level of sequence conservation points to a recent horizontal gene transfer between these two species possibly mediated by the natural *Corynebacterium* plasmid pLEW279b. This plasmid contains a gene region with 97% nucleotide similarity to the *cop* gene region of *C. glutamicum* wild type. It was shown that the plasmid sequence is a mosaic of genes and accessory elements acquired from related Gram-positive sources [28].

In *C. diphtheriae* a gene encoding a small protein of about 9 kDa was proposed to be located between the homologues of cg3286 and *copR*. In *C. glutamicum* the nucleotide sequence of this gene can also be found within the intergenic region of cg3286 and *copR* extending from position −108 to −334 bp with respect to the *copR* translational start site and orientated divergently to *copR*. The translational start point of the hypothetical gene would be located 30 bp upstream of the CopR binding site and might form an operon structure with the following cg3286 gene. However, so far no evidence is available that this putative gene is expressed in *C. diphtheriae* or *C. glutamicum*. Another explanation for the long distance between the CopR binding site and the cg3286 transcriptional start site could be the existence of a regulatory RNA element within the intergenic region, but inspection of this region with the software tool RNAstructure [29] revealed no significant RNA structure. Further experiments are on the way to assign a function to this DNA region.

The copper stimulon not only consists of the *cop* region (cg3281-cg3289), but also of genes coding for proteins involved in heme biosynthesis and cytochrome *c* maturation (Table 1). It can be assumed that excess of copper stimulates synthesis of heme and cytochrome c since these are also cofactors of the respiratory chain protein complexes as well as copper. A link between iron and copper was previously also shown for *E. coli* [30], *P. aeruginosa* [26], [31], yeasts [32] and mammals [33]. The existence of this mechanistic link in *C. glutamicum* is further underlined by the finding that the putative copper transporter CtpV (Cg0464; Accession UniProtKB Q6M7X6) belongs to the regulon of the master regulator of iron homeostasis, DtxR [34]. The *ctpV* gene together with the downstream located gene *csoR* were highly upregulated in the presence of elevated copper ion concentrations (1091-fold (*P*-value of 0.07) and 9-fold (*P*-value of 0.06) changed mRNA level, respectively) in *C. glutamicum* wild type. *csoR* codes for a homologue of the transcriptional regulator CsoR from *M. tuberculosis* (27% identity) which was shown to be involved in the copper stress response [12] by binding intracellular Cu^+^ followed by derepression of the *ctpV* gene coding for a putative copper-exporting P-type ATPase [13]. Therefore, it is reasonable to assume that CsoR (Accession UniProtKB Q8NTC2) of *C. glutamicum* also functions as a cytoplasmatic copper sensor and regulates expression of *ctpV*. Studies are underway to confirm this hypothesis. Taken together, our work suggests that *C. glutamicum* possesses both an intracellular copper sensor, i. e. CsoR, and the extracellular copper sensor system CopRS.

**Figure 7:**
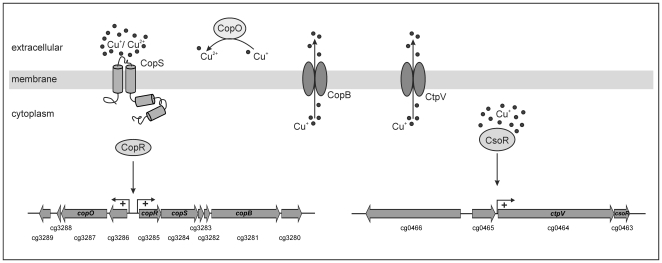
Model of copper excess response in *C. glutamicum*. The CopS sensor kinase recognises high extracellular copper concentrations followed by autophosphorylation and phosphotransfer to the response regulator CopR. Phosphorylated CopR binds to the direct repeat (TGAAGATTTnnTGAAGATTT) within the cg3286-*copR* intergenic region. This results in a transcriptional activation of both putative operons (cg3286-cg3289 and *copR*-cg3281) containing genes encoding copper resistance proteins, e.g. a putative multicopper oxidase (CopO) and a copper export ATPase (CopB). CopO can detoxify Cu^+^ by converting it to the less toxic Cu^2+^ and by binding free Cu ions. CopB is a cation ATPase and likely functions as a copper export pump. The Cu-specific regulator CsoR senses high intracellular copper concentrations and activates (or derepresses) the transcription of the copper export ATPase CtpV which is part of the copper detoxification process.

Based on these data we can propose a model of the dual copper resistance mechanism in *C. glutamicum* (Fig. 7). Under aerobic conditions copper is present in the periplasmic space in the Cu^+^ and Cu^2+^ state [35]. The two-component system CopRS recognises high extracellular copper concentrations followed by transcriptional activation of the two putative operons cg3286-cg3289 and *copR*-cg3281 containing genes encoding copper resistance proteins, e.g. the putative multicopper oxidase CopO and the putative copper export ATPase CopB. As a result CopB exports excess of Cu^+^ from the cytoplasm to the ‘periplasm’. In the ‘periplasm’ CopO can detoxify Cu^+^ by oxidising it to Cu^2+^ which is less toxic and less able to diffuse through the cytoplasmic membrane [7] and by sequestering free Cu ions. The Cu-specific regulator CsoR senses high intracellular copper concentrations and activates (or derepresses) transcription of the copper export ATPase CtpV which also might be responsible for exporting excess copper from the cytoplasm.

## Materials and Methods

### Bacterial strains, media, and growth conditions

Bacterial strains and plasmids used or constructed in the course of this work are listed in Table 3, oligonucleotides in supplementary Table S1. *C. glutamicum* was routinely cultivated aerobically in 500-ml shaking flasks with 50 ml CGXII minimal medium [36] containing 4% (w/v) glucose as carbon and energy source on a rotary shaker (120 rpm) at 30°C. For strain construction and maintenance, BHIS agar plates (BHI agar (Difco, Detroit, USA) with 0.5 M sorbitol) were used. *E. coli* DH5α was grown aerobically on a rotary shaker (120 rpm) at 37°C in LB medium or on LB agar plates (LB medium with 1.5% (w/v) agar). If appropriate, kanamycin was added to final concentrations of 25 µg/ml (*C. glutamicum*) or 50 µg/ml (*E. coli*).

**Table 3:** Strains and plasmids used in this study.

Strain or plasmid	Relevant characteristics	Source or reference
**Strains**		
*C. glutamicum* ATCC13032	Biotin-auxotrophic wild type strain	[51]
*C. glutamicum* Δ*copRS*	Derivative of ATCC13032 with an in-frame deletion of the *copRS* genes	[21]
*E. coli* DH5α	F^-^ φ80*dlac*Δ (*lacZ*)M15 Δ (*lacZYA-argF*) U169 *endA1 recA1 hsdR17* (r_K_^-^, m_K_^+^) *deoR thi-1 phoA supE44* λ^-^ *gyrA96 relA1*	Invitrogen
*E. coli* BL21(DE3)	*ompT hsdS*_B_ (r_B_^-^m_B_^-^) *gal dcm* (DE3)	[52]
**Plasmids**		
pET28b	Kan^r^; vector for overexpression of genes in *E. coli*, adding an N-terminal hexahistidine affinity tag to the synthesised protein (pBR322 *oriV_E.c._ P_T7_ lacI*)	Novagen, Merck KGaA
pET28b-NHis_6_-CopR	Kan^r^; pET28b derivative for overproduction of CopR with an N-terminal hexahistidine tag	This work
pEKEx2	Kan^r^; *C. glutamicum/E. coli* shuttle vector for regulated gene expression (*P_tac_*, *lacI* ^Q^, pBL1 *oriV_C. g_*_._, pUC18 *oriV_E. c._*)	[39]
pEKEx2-*copRS*	Kan^r^; pEKEx2 derivative containing the *copRS* genes from *C. glutamicum* under control of the *tac* promoter	This work
pET2	Kan^r^; promoter-probe vector	[40]
pET2-IGR	Kan^r^; pET2 with the whole intergenic region of cg3286 and *copR* (402 bp), orientated cg3286 to *copR*	This work
pET2-IGR_inverse	Kan^r^; pET2 with the whole intergenic region of cg3286 and *copR* (402 bp), orientated *copR* to cg3286	This work

### Agar diffusion assay

To compare the resistance of *C. glutamicum* to heavy metal ions, strains were grown in CGXII minimal medium to an OD_600_ of 6. The resulting cells were diluted to an OD_600_ of 0.05 in CGXII soft agar (CGXII medium with 0.75% (w/v) agar) and 4 ml thereof were poured onto a CGXII agar plate. Once the agar became solid, a glass fibre filter disc (10 mm diameter, 2 µm pore size; Millipore) was placed onto the plate and 70 µl of a heavy metal ion solution (different concentrations of CuSO_4_, NiSO_4_, MnCl_2_, ZnCl_2_, AgNO_3_, CoCl_2_, PbCl_2_ or CdCl_2_) were spotted onto the paper disc. The plates were incubated at 30°C and the zones of inhibition were determined after 48 h. If appropriate, kanamycin (25 mg/ml) and IPTG (1 mM) were added to the media.

### Recombinant DNA work

The enzymes for recombinant DNA work were obtained from Roche Diagnostics or New England Biolabs. All oligonucleotides were synthesised by Eurofins MWG Operon (Table S1). Routine methods like PCR, restriction or ligation were carried out according to standard protocols [37]. Plasmids were isolated from *E. coli* with the QIAprepspin miniprep kit (Qiagen). *E. coli* was transformed by the RbCl method [38]. All constructs described below were confirmed via DNA sequencing performed by LGC genomics.

### Construction of expression plasmids

For IPTG-inducible expression of *copRS*, the coding region of these genes including 9 bp upstream of the *copR* start codon plus an artificial ribosomal binding site (AAGGAGA) was amplified using chromosomal DNA of *C. glutamicum* ATCC 13032 as template, the oligonucleotide pair copRS_SalI_fw/copRS_EcoRI_rv and the Expand High Fidelity PCR kit (Roche Diagnostics). The PCR product was digested with SalI and EcoRI and cloned into pEKEx2 [39] cut with the same enzymes.

For overproduction and purification of CopR with an amino-terminal histidine tag, the *copR* coding region was amplified using oligonucleotides that introduce an NdeI restriction site (copR_NdeI_fw) and a XhoI restriction site after the stop codon (copR_XhoI_rv). The purified 740 bp PCR product was digested with NdeI and XhoI and cloned into the expression vector pET28b, resulting in plasmid pET28b-NHis_6_-CopR. The CopR protein encoded by this plasmid (260 amino acids, 28.8 kDa) contains 20 additional amino acids (MGSSHHHHHHSSGLVPRGSH) at the N-terminus including a hexahistidine tag and a thrombin cleavage site.

### Construction of reporter gene plasmids and measurement of chloramphenicol acetyltransferase activity

The whole intergenic region, meaning the region between the start codons of the genes cg3286 and *copR* (402 bp), was amplified with the primer pairs PstI_fw IGR-cg3286_copR/BamHI_rv IGR-cg3286_copR and BamHI_fw IGR-cg3286_copR/PstI_rv IGR-cg3286_copR, respectively, and cloned into the corynebacterial promoter-probe vector pET2 [40]. The resulting plasmids pET2-IGR and pET2-IGR_inverse contained the intergenic region upstream of the promoterless *cat* gene and were transferred into *C. glutamicum* wild type and the Δ*copRS* mutant by electroporation. The promoter activities were determined by measuring the chloramphenicol acetyltransferase (CAT) activity via the formation of 5-thio-2-nitrobenzoate photometrically at 412 nm and 30°C [41]–[43].

### DNA microarray analysis

For RNA preparation, *C. glutamicum* strains were cultivated overnight in CGXII minimal medium containing 4% (w/v) glucose. Cells from these precultures were washed in 0.9% (w/v) NaCl and used for inoculation of CGXII minimal medium containing 4% (w/v) glucose with or without the addition of 20 µM CuSO_4_. At an OD_600_ of 5–6, 20 ml of the cultures were poured into ice-containing tubes precooled to −20°C and cells were harvested by centrifugation (3 min, 4200 x *g*, 4°C). The cell pellet was directly used for RNA isolation as described before [44]. All DNA microarray analyses were performed with custom-made DNA microarrays based on 70-mer oligonucleotides obtained from Operon Biotechnologies. The comparisons were performed from four or five independent biological replicates. The experimental details for handling of these microarrays were described before [45]. We defined the experimental data as significant if the mRNA level showed an at least threefold change in at least two of four (Table 1) or three of five (Table 2) replicates and the *P*-value was ≤0.05. Processed and normalized data as well as experimental details conformed to the MIAME guidelines [46] were stored in the in-house microarray database [47] for further analysis and in the Gene Expression Omnibus (GEO) repository under the accession number GSE27510.

### Primer extension analysis

Nonradioactive primer extension analyses of cg3286 were performed as described previously [48], [49] using the IRD800-labeled oligonucleotides PE_cg3286_30 and PE_cg3286_80 (Table S1) and 10 µg RNA from *C. glutamicum* wild type or strain Δ*copRS* as template. The length of the primer extension product was determined by running the four lanes of a DNA-sequencing reaction mixture set up using the same oligonucleotide as that used for the reverse transcription alongside the primer extension product. The template for DNA sequencing covered the whole promoter region and was obtained by PCR using chromosomal DNA of *C. glutamicum* ATCC13032 and the oligonucleotide pair cg3286_fw/cg3286_rv (Table S1).

### Overproduction and purification of CopR

The response regulator CopR was overproduced in *E. coli* BL21(DE3) using the expression plasmid pET28b-NHis_6_-CopR. Cells were grown at 37°C and 120 rpm in 500 ml LB medium with 50 µg/ml kanamycin to an OD_600_ of ∼0.6 before adding 1 mM isopropyl *β*–D-thiogalactoside (IPTG). After cultivation for another 4 hours, cells were harvested by centrifugation at 4°C and stored at −20°C. NHis_6_-CopR was purified by Ni^2+^-chelate affinity chromatography followed by buffer exchange against bandshift buffer (50 mM Tris-HCl, 50 mM KCl, 10 mM MgCl_2_, 0.5 mM EDTA, 10% (v/v) glycerol, pH 7.5) as described for the response regulator CitB previously [44]. Protein concentrations were determined with the Bradford protein assay (Uptima) using bovine serum albumin (BSA) as standard.

### Electrophoretic mobility shift assays (EMSAs)

For testing the binding of CopR to putative target promoters, purified CopR protein (0–18 µM) was mixed with 100 ng DNA fragments (30–229 bp, final concentration 25–255 nM) in a total volume of 20 µl. The binding buffer contained 50 mM Tris-HCl (pH 7.5), 50 mM KCl, 10 mM MgCl_2_, 0.5 mM EDTA and 10% (v/v) glycerol. NHis_6_-CopR, either in the unphosphorylated state or after phosphorylation with 50 mM acetylphosphate, was mixed with the DNA fragment, incubated at room temperature for 30 min and then loaded onto a 10% (DNA fragments >100 bp) or 15% (DNA fragments <100 bp) native polyacrylamide gel. For phosphorylation of NHis_6_-CopR, the protein was incubated for 60 min with 50 mM acetylphosphate before addition of the DNA fragments. Electrophoresis and DNA detection with SybrGreen I were performed as described before [50]. The DNA fragments were generated by PCR or by primer annealing for insertion of base pair mutations, purified with the PCR purification Kit (Qiagen) and eluted in double-distilled H_2_O. For determination of the apparent *K_d_* value, the DNA fragment (90 bp) was amplified using an oligonucleotide labelled with the fluorescent dye Cy3 (Eurofins MWG Operon). Purified and phosphorylated CopR was mixed with the DNA fragment (8 nM), incubated at room temperature for 30 min and then loaded onto a 15% native polyacrylamide gel. Electrophoresis was performed as described before [50]. The DNA was detected using a fluorescence scanner (Typhoon Trio, GE Healthcare) at an excitation of 532 nm and an emission of 580 nm.

## Supporting Information

Figure S1 **EMSAs for testing the binding of CopR.** The binding of CopR to the promoter regions of putative target genes referring to Table 2 was analysed. Fragment cg1665 served as negative control. DNA fragments (100 ng, 157–263 bp) were incubated for 30 min at room temperature with phosphorylated CopR at different molar excesses (0- to 100-fold, see legend). For experimental details see Material and Methods.
(TIF)

Figure S2 **Electrophoretic mobility shift assays and calculated plots for the determination of the apparent** ***K_d_*** **values.** The *K_d_* values of unphosphorylated CopR (A) and phosphorylated CopR (B) binding to the *copR* promoter region were determined. The 5′-Cy3-labelled DNA probes (8 nM) were incubated with various amounts of phosphorylated (0–500 nM) and unphosphorylated (0–5000 nM) CopR, respectively. Free and CopR-bound DNA were separated by electrophoresis using a 15% native polyacylamide gel and detected using a fluorescence scanner. The amount of free and protein-bound DNA was quantified using ImageQuant™ TL (GE Healthcare). The ratios of the amount of bound to total DNA were calculated and plotted against the protein concentration in order to determine the *K_d_* values.
(TIF)

Figure S3 **Primer extension analysis of the gene cg3286.** The analysis was performed using the oligonucleotide PE_cg3286_30 and 10 µg of total RNA from wild type and the Δ*copRS* mutant. The transcriptional start site is indicated by an asterisk. The strains were grown in CGXII medium with 4% (w/v) glucose supplemented with 20 µM CuSO_4_ and harvested in the exponential growth phase for RNA isolation.
(TIF)

Table S1 **Oligonucleotides used in this study.**
(DOC)
