# Supplementary figures and images for: The Two-Component Signal Transduction System CopRS of Corynebacterium glutamicum Is Required for Adaptation to Copper-Excess Stress
Source: PLoS One. 2011 Jul 20;6(7):e22143. doi: 10.1371/journal.pone.0022143 (PMC3140484; doi:10.1371/journal.pone.0022143)

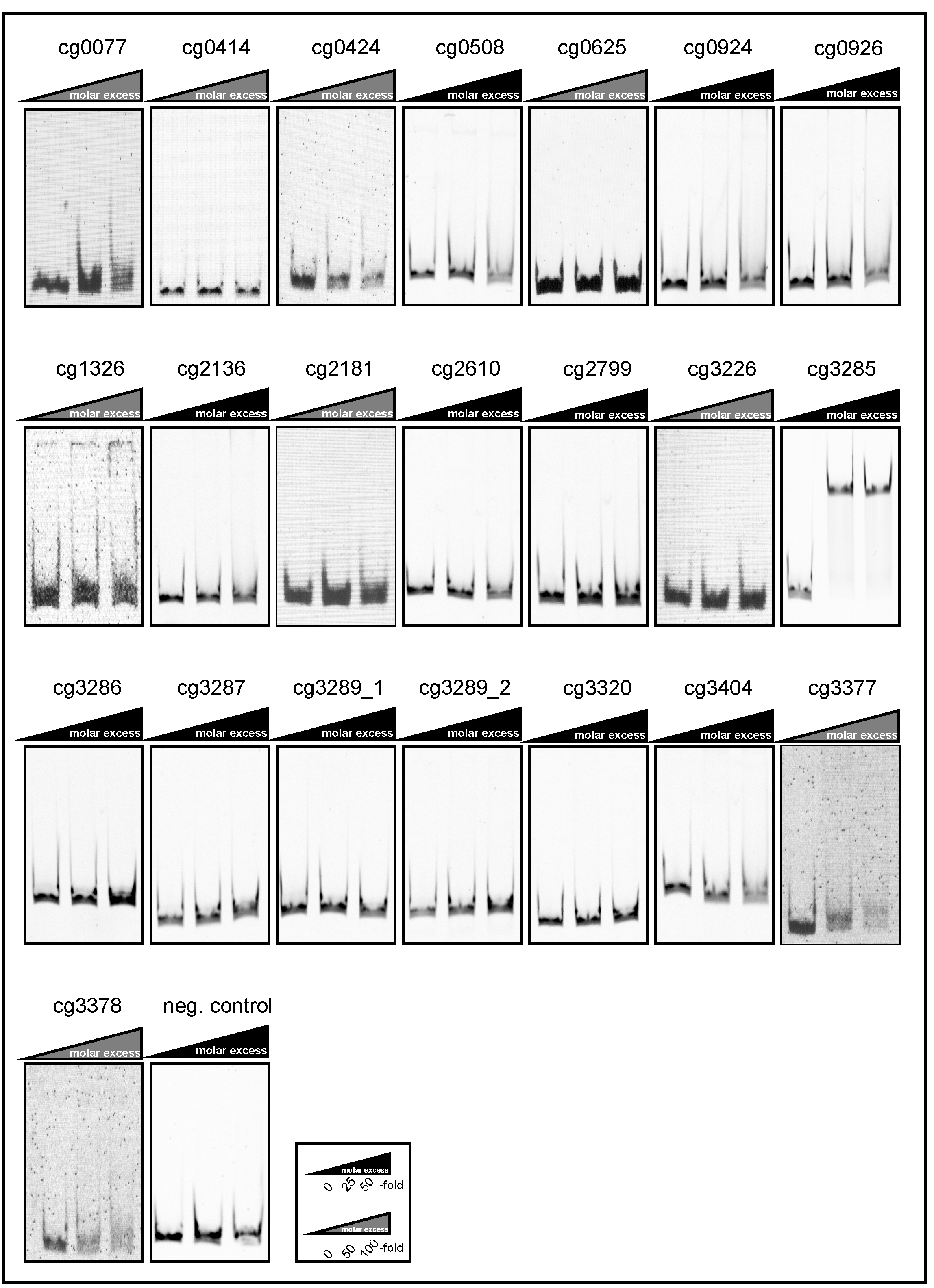

Supplement: Figure S1 — EMSAs for testing the binding of CopR. The binding of CopR to the promoter regions of putative target genes referring to Table 2 was analysed. Fragment cg1665 served as negative control. DNA fragments (100 ng, 157–263 bp) were incubated for 30 min at room temperature with phosphorylated CopR at different molar excesses (0- to 100-fold, see legend). For experimental details see Material and Methods. (TIF) [file pone.0022143.s001.tif]

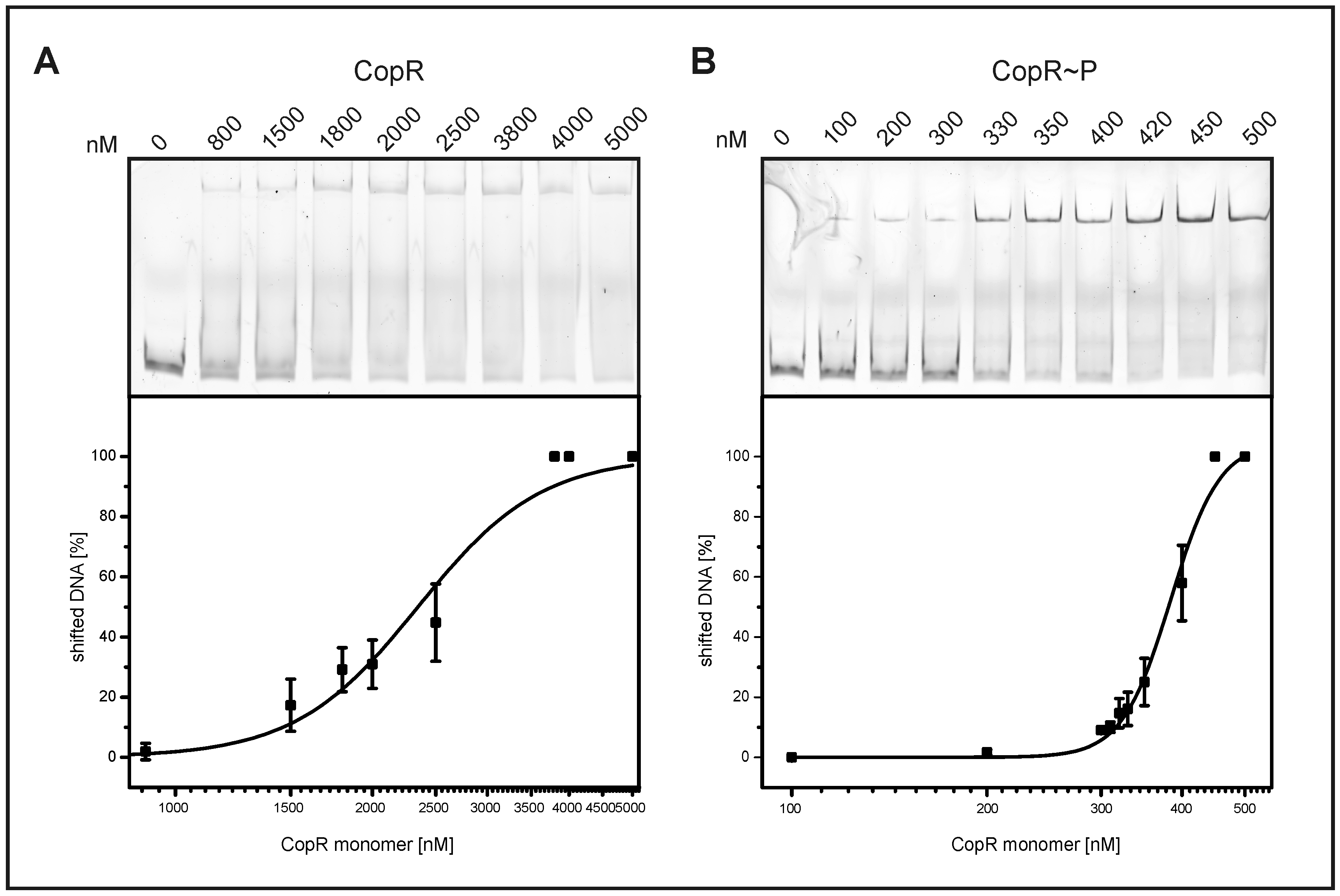

Supplement: Figure S2 — Electrophoretic mobility shift assays and calculated plots for the determination of the apparent Kd values. The Kd values of unphosphorylated CopR (A) and phosphorylated CopR (B) binding to the copR promoter region were determined. The 5′-Cy3-labelled DNA probes (8 nM) were incubated with various amounts of phosphorylated (0–500 nM) and unphosphorylated (0–5000 nM) CopR, respectively. Free and CopR-bound DNA were separated by electrophoresis using a 15% native polyacylamide gel and detected using a fluorescence scanner. The amount of free and protein-bound DNA was quantified using ImageQuant™ TL (GE Healthcare). The ratios of the amount of bound to total DNA were calculated and plotted against the protein concentration in order to determine the Kd values. (TIF) [file pone.0022143.s002.tif]

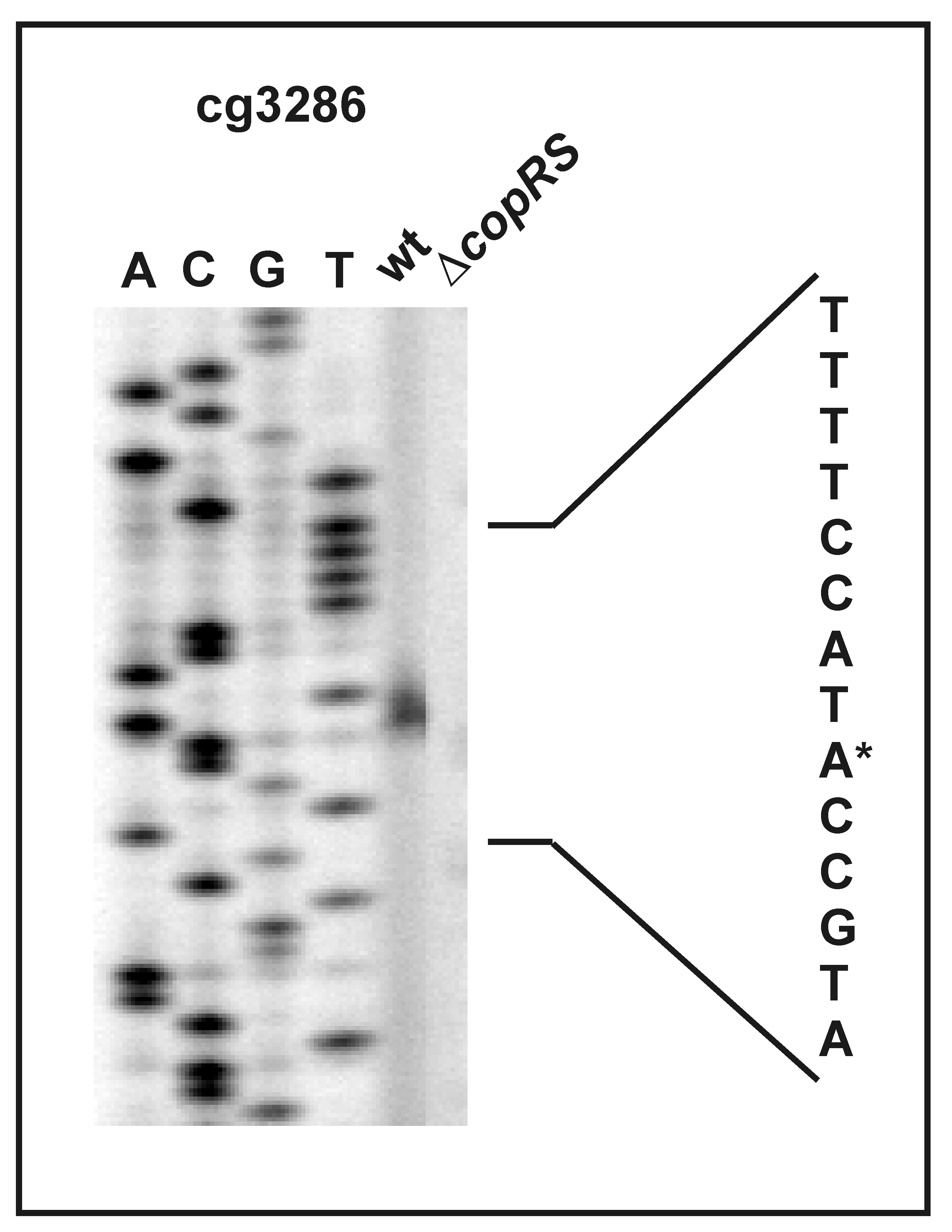

Supplement: Figure S3 — Primer extension analysis of the gene cg3286. The analysis was performed using the oligonucleotide PE_cg3286_30 and 10 µg of total RNA from wild type and the ΔcopRS mutant. The transcriptional start site is indicated by an asterisk. The strains were grown in CGXII medium with 4% (w/v) glucose supplemented with 20 µM CuSO4 and harvested in the exponential growth phase for RNA isolation. (TIF) [file pone.0022143.s003.tif]
