# Supplementary material for: The Two-Component Signal Transduction System CopRS of Corynebacterium glutamicum Is Required for Adaptation to Copper-Excess Stress
Source: PLoS One. 2011 Jul 20;6(7):e22143. doi: 10.1371/journal.pone.0022143 (PMC3140484; doi:10.1371/journal.pone.0022143)
Supplement: Table S1 — Oligonucleotides used in this study. (DOC) [file pone.0022143.s004.doc]

**Table S1. Oligonucleotides used in this study.**

| **Oligonucleotide** | **Sequence (5’ → 3’) and properties^a^** |
| --- | --- |
| **Construction of pEKEx2-*copRS*** | |
| cgtSR9_SalI_fw | TAT AGT CGA C**AA GGA GA**C TGG GGT CTA TGG CTG ACC GC (SalI) |
| cgtSR9_EcoRI_rv | TAT AGA ATT CCC CTA TGG GGT ATT GTC AAG GG (EcoRI) |
| **Construction of pET28b-NHis_6_-CopR** | |
| cgtR9_NdeI_fw | TAT ACA TAT GGC TGA CCG CAC ACC GAC C (NdeI) |
| cgtR9_XhoI_rv | TAT ACT CGA GTC ATG GCT GCC CCA CCC GGT AG (XhoI) |
| **Construction of pET2-IGR** | |
| PstI_fw IGR-cg3286_copR | TAT ACT GCA GGT CAA TCT CCT TGG ATC GTG G (PstI) |
| BamHI_rv IGR-cg3286_copR | TAT AGG ATC CAG ACC CCA GTA TCG CTT CGG (BamHI) |
| **Construction of pET2-IGR_inverse** | |
| BamHI_fw IGR-cg3286_copR | TAT AGG ATC CGT CAA TCT CCT TGG ATC GTG G (BamHI) |
| PstI_rv IGR-cg3286_copR | TAT ACT GCA GAG ACC CCA GTA TCG CTT CGG (PstI) |
| **Primer extension** |  |
| PE_cg3286_30 | IRD800- CAC CCC GTG AGG GCG AGG GTA A |
| PE_cg3286_80 | IRD800- ATC CTG GGA CAC CGT CCC GTC |
| cg3286_fw | GTG GGA CGC TCA GTG ACC TTC TG |
| cg3286_rv | TCA TCG ACG ACC AGC ACC CGC |
| **EMSAs** |  |
| cg0077_fw | CCC CAT CTT CTA AAC AAG AAA GG |
| cg0077_rv | CGT TTG TAC GAA TGA CTG GCT G |
| cg0414-fw | CAC CAC CCC TCT CAA ATA GCC |
| cg0414-rv | GAA GCT CAG GTT CCT TAA TTT AG |
| cg0424_fw | GTC ATA TTC TGG TGA TTT TCG CC |
| cg0424_rv | GAT GCA TCA TCA AAA ACA ATT AAT TC |
| cg0508-fw | TCT TTT CCC TAC CCA CAT AAC CC |
| cg0508-rv | GAA GAC ATG AAA ATA AGG CCC TTC |
| P_NCgl0513_fw_2 | GCA ATC GCT TTC ACC AGG TAT TG |
| P_NCgl0513_rv_2 | CAG ACC AGC GAA TGG TTC TCT TC |
| cg0924-fw | AAA GTG CCA AAT TAG ACA GGT TTT C |
| cg0924-rv | CAC CAT GTC GTC CTT TGA AAG TC |
| cg0926-fw | GAT CGA TCC CAC ACT GAC CCC |
| cg0926-rv | CCA TCA GTG CCG ATC CCC TTC |
| P_NCgl1127_fw_1 | TTC ATG TTC ATC TTT CTC AAC AAT C |
| P_NCgl1127_rv_1 | GTG ACA GGC TAT TCT AAA CTG ATA G |
| cg2136-fw | GGC TCG TCT CCG ACC TTG CC |
| cg2136-rv | GTC ATA AGG GTA GCT TAA AGC AG |
| cg2181_fw | CCT AGC AAC TTA GTT AAT TTT CCC |
| cg2181_rv | CAA AGC AAG TGC AGC CGC CG |
| cg2610-fw | GCA TGA ACT TGA CCC TAG CAG C |
| cg2610-rv | GCG CAT TAA ATC TCC TAT GGG TG |
| cg2799-fw | TGG TCA GCG AAG TAC TCG GAC |
| cg2799-rv | TCG CAG GGG GCG ATC ATT CC |
| cg3226_fw | GTA ATT GGA TTC GAC TGT TTT CC |
| cg3226_rv | CAG GAC GCG CTT GTT GAG TTG |
| P_NCgl2863_fw_1 | CGT GGA CGA TGT CCT TCT ACC C |
| NCgl2863_rv3 | CAG TGC CCG GCG GGG AAA TC |
| NCgl2863_fw4 | GAT TTC CCC GCC GGG CAC TG |
| NCgl2863_rv4 | GTC GAA GCC GGC CCG GAT G |
| BS9A2-fw | GGG TGA TGT TTG AAG ATT TGA TG |
| BS9A1-rv | CAT CAA ATC TTC AAA CAT CAC CC |
| P_NCgl2864_fw_1 | GGG TAG AAG GAC ATC GTC CAC G |
| P_NCgl2864_rv_1 | CGC TGC TCG TTT CAC GTC AAT CTC |
| P_NCgl2865_fw_1 | GAC GGG GCC ACC TGT GTC AC |
| P_NCgl2865_rv_1 | CCG GTG CCG GCG AGT ACG AG |
| P_NCgl2866_fw_1 | GCT CGC TTC GCT ACG AGT AAC C |
| P_NCgl2866_rv_1 | GCA CCG CGG CAA CCG CGA TG |
| P_NCgl2866_fw_2 | CAT CGC GGT TGC CGC GGT GC |
| P_NCgl2866_rv_2 | CAG ACA GGC TGA TCT CCT CAC C |
| cg3320-fw | GTC AGA AGC AGC GCG TGG CC |
| cg3320-rv | GCA AGG CTC ATG AGT GTT TCA C |
| cg3404-fw | AGT GTT GTG AGT GAT TGC GCG C |
| cg3404-rv | CGA ATA GAT TGC ATT AGG CTA TCC |
| cg3377_fw | CAT CCC CCA ATG CGA TCC GG |
| cg3377_rv | CAA ACA TAG TGA TCT CCT TAA AGT G |
| cg3378_fw | GGC ACA CCT TGC AAG TAC ACG |
| cg3378_rv | CAG GCT GGA AGT TTT GTG CAA TG |
| **Cy3 labelled DNA for K_d_ determination** | |
| Cy3-overlap-NCgl2863_fw2 | ACA TCA CGC ACG TAC CCA TTT CGC ACA GGG GTG ACG GCG GTC G |
| NCgl2863_rv3 | CAG TGC CCG GCG GGG AAA TC |
| Cy3-labelled_primer | ACA TCA CGC ACG TAC CCA TTT CG |
| **CopR binding motif mutations** | |
| BM-Mut1_fw | ATG GCG TAA GAT TTG ATG AAG ATT TCC CCG |
| BM-Mut1_rv | CGG GGA AAT CTT CAT CAA ATC TTA CGC CAT |
| BM-Mut2_fw | ATG TTT GCG TAT TTG ATG AAG ATT TCC CCG |
| BM-Mut2_rv | CGG GGA AAT CTT CAT CAA ATA CGC AAA CAT |
| BM-Mut3_fw | ATG TTT GAA GGC GTG ATG AAG ATT TCC CCG |
| BM-Mut3_rv | CGG GGA AAT CTT CAT CAC GCC TTC AAA CAT |
| BM-Mut4_fw | ATG TTT GAA GAT TCT GCG AAG ATT TCC CCG |
| BM-Mut4_rv | CGG GGA AAT CTT CGC AGA ATC TTC AAA CAT |
| BM-Mut5_fw | ATG TTT GAA GAT TTG ATA GCG ATT TCC CCG |
| BM-Mut5_rv | CGG GGA AAT CGC TAT CAA ATC TTC AAA CAT |
| BM-Mut6_fw | ATG TTT GAA GAT TTG ATG AAA CGT TCC CCG |
| BM-Mut6_rv | CGG GGA ACG TTT CAT CAA ATC TTC AAA CAT |
| BM-Mut7_fw | ATG TTT GAA GAT TTG ATG AAG ATC GTA CCG |
| BM-Mut7_rv | CGG TAC GAT CTT CAT CAA ATC TTC AAA CAT |
| BM-Mut8_fw | ATG GCT GAA GAT TTG ATG AAG ATT TCC CCG |
| BM-Mut8_rv | CGG GGA AAT CTT CAT CAA ATC TTC AGC CAT |
| BM-Mut9_fw | ATG TTG GAA GAT TTG ATG AAG ATT TCC CCG |
| BM-Mut9_rv | CGG GGA AAT CTT CAT CAA ATC TTC CAA CAT |
| BM-Mut10_fw | ATG TTT GAA GGT TTG ATG AAG ATT TCC CCG |
| BM-Mut10_rv | CGG GGA AAT CTT CAT CAA ACC TTC AAA CAT |
| BM-Mut11_fw | ATG TTT GAA GAT TTT GTG AAG ATT TCC CCG |
| BM-Mut11_rv | CGG GGA AAT CTT CAC AAA ATC TTC AAA CAT |
| BM-Mut12_fw | ATG TTT GAA GAT TTG ATG AAA ATT TCC CCG |
| BM-Mut12_rv | CGG GGA AAT TTT CAT CAA ATC TTC AAA CAT |
| BM-Mut13_fw | ATG TTT GAA GAT TTG ATG AAG ATC GCC CCG |
| BM-Mut13_rv | CGG GGC GAT CTT CAT CAA ATC TTC AAA CAT |
| BM-Mut14_fw | ATG TTT GAA GAT TTG ATG AAG ATT TTA CCG |
| BM-Mut14_rv | CGG TAA AAT CTT CAT CAA ATC TTC AAA CAT |
| BM-Mut15_fw | ATA CGT GAA GAT TTG ATG AAG ATT TCC CCG |
| BM-Mut15_rv | CGG GGA AAT CTT CAT CAA ATC TTC ACG TAT |
| BM-Mut16_fw | ATG TTG TAA GAT TTG ATG AAG ATT TCC CCG |
| BM-Mut16_rv | CGG GGA AAT CTT CAT CAA ATC TTA CAA CAT |
| BM-Mut17_fw | ATG TTT GAA GAC GTG ATG AAG ATT TCC CCG |
| BM-Mut17_rv | CGG GGA AAT CTT CAT CAC GTC TTC AAA CAT |
| BM-Mut18_fw | ATG TTT GAA GAT TTG ATA GAG ATT TCC CCG |
| BM-Mut18_rv | CGG GGA AAT CTC TAT CAA ATC TTC AAA CAT |
| BM-Mut19_fw | ATG TTT GAA GAT TTG ATG AAA CTT TCC CCG |
| BM-Mut19_rv | CGG GGA AAG TTT CAT CAA ATC TTC AAA CAT |

^a^ In some cases oligonucleotides were designed to introduce recognition sites for restriction endonucleases (recognition sites underlined, restriction endonucleases indicated in parentheses), and ribosomal binding sites (printed in bold) into the resulting PCR products.
